# Effect of Thermodynamic Cyclic Loading on Screw Loosening of Tightened Versus New Abutment Screw in Bone Level and Tissue Level Implants in DIO Implant Company (In‐Vitro Study)

**DOI:** 10.1002/cre2.70162

**Published:** 2025-06-27

**Authors:** Amirhossein Fathi, Sina Borhani, Sepideh Salehi, Ramin Mosharraf, Ramin Atash

**Affiliations:** ^1^ Dental Prosthodontics Department, School of Dentistry, Dental Materials Research Center Isfahan University of Medical Sciences, Isfahan, Iran Isfahan Iran; ^2^ School of Dentistry Isfahan University of Medical Sciences, Isfahan, Iran Isfahan Iran; ^3^ Department of Dentistry Free University of Brussels (ULB) Brussels Belgium

**Keywords:** cyclic dynamic loading, dental implants, screw loosening

## Abstract

**Objectives:**

The loosening of abutment screws is a common mechanical complication in dental implants, potentially leading to treatment failure. It is generally believed that dynamic forces may accelerate and facilitate screw loosening. Two approaches exist to address this issue: replacing the screw or re‐tightening the existing one. However, there is no consensus on the efficacy of either method in preventing re‐loosening.

**Material and Methods:**

In this laboratory study, 20 implant‐abutment sets with a diameter of 4.5 mm were randomly placed in resin blocks simulating bone tissue. The samples were tightened with a torque of 30 N cm and subjected to 10,000 dynamic cycles in a thermocycler device at temperatures ranging from 5°C to 55°C because it is the usual temperature of the mouth while drinking or eating cold and hot things. After dynamic cycling, the torque required to unscrew the abutment screws was measured using a digital torque meter, and the torque reduction in both groups was calculated.

**Results:**

The results showed a significant reduction in the torque of abutment screws in both implant types after dynamic loading. Additionally, tissue‐level implants experienced greater loosening than Bone‐level implants, requiring less force to unscrew the abutment in the Tissue‐level group. New screws showed less torque reduction compared to previously tightened screws. Opening torque scores in the new bone‐level implants after applying the primary thermodynamic cycle (26.7 N cm) were more than in the new tissue‐level implants (24.7 N cm). The torque reduction percentage values of the implant after the primary thermodynamic cycle were significantly higher in the tissue‐level (17.67 N cm) group than in the bone‐level (11 N cm) group.

**Conclusions:**

Overall, dynamic loading can cause loosening of abutment screws in dental implants, with Tissue level implants being more susceptible. It is recommended to periodically replace screws with new ones to prevent torque reduction and related complications.

## 1 Introduction

With the emergence of dental implants, a great revolution occurred in the field of dental prosthesis science, and often, osseointegrated implants are regarded as an extremely successful treatment for edentulous and partially edentulous patients. However, this treatment may bring up problems related to the biological and mechanical aspects of it. The first and the biological obstacle that the surgeon must consider is the lack of osseointegration. However, even assuming that osseointegration happens properly, the prosthodontist, the general dentist, and the laboratory technician will face mechanical obstacles trying to make an implant‐supported prosthesis. Dental implants consist of the 3 parts of fixture, abutment, and crown, and the responsibility of the fixture‐abutment junction is carried out by the abutment screw. Since the abutment screw is one of the weakest parts of the implant, the biggest mechanical challenge that the dentist and the technician have to deal with is the loosening or fracture of the abutment screw (E. Jung et al. 2012).

Screw loosening occurs when the forces separating screw connections exceed the retentive forces that keep the screw unit together. While tightening the screw, the microscopic roughnesses of the metallic contact surface smoothen, and the gap between the contact surfaces reduces (Krishnan et al. 2014). Various factors have been considered effective in the incidence of screw loosening, of which we can mention the design and dimensions of the restoration, the incompatibility of the prosthesis and the abutment, or the abutment and the implant, the insufficient force of screw tightening (McAlarney and Stavropoulos 1996), biomechanical overload (Haack et al. 1995), the material and design of the screw (Kallus and Bessing 1994), the bite force of the patient and the lack of parafunctional habits (McAlarney and Stavropoulos 1996), and the level of accuracy in the connection of the attachments. Different studies have been conducted to investigate those factors, however, there is a research gap investigating the effect of thermodynamic cyclic loading on screw loosening in implants. So, in this study, we try to fill this gap.

Incompatibility can exist between the components of a system or the combination of the components of different systems. Studies have shown that the more reliable the level of compatibility between the components of a system, the less the chance of problems caused by incompatibility (Byrne et al. 2006; Hebel and Gajjar 1997; Johansson and Ekfeldt 2003). On the other hand, some companies have intended to create components similar to the main components of the current systems. The purpose behind the creation of these components is to provide more options when choosing the abutment, especially when the main components are not available, and to reduce expenses. The important points in the use of these components are the success rate, prosthetic treatment durability, and their level of compatibility. Unlike the claims of the companies about the total compatibility of these parts with the main components, it is necessary to assess their level of compatibility in various investigations, because a lack of accurate compatibility between the implant components and the abutment leads to several biological and mechanical problems (Jansen et al. 1997; Vidigal GM et al. 1995; Al‐Turki et al. 2002).

Implant treatment can cause many complications. These complications generally include peri‐mucositis, peri‐implantitis, loosening or fracture of the abutment screw, fracture of the abutment or the superstructure, loosening of the crown, or fracture of the porcelain (E. Jung et al. 2012; Wittneben et al. 2014; Sahin and Ayyildiz 2014; Goodacre et al. 2003; Zembic et al. 2014). Between these factors, the loosening of the abutment screw is one of the most common mechanical complications (Goodacre et al. 2003; Zembic et al. 2014; Assenza et al. 2005; Assunção et al. 2012). Another meaning of “preload” is the axial force produced between the abutment screw threads and the internal parts of the implant in the direction of the longitudinal axis of the implant (Assunção et al. 2011; Wang et al. 2009; Burguete et al. 1994; Cantwell and Hobkirk 2004). The preload force must continue and be minimized to prevent the separation of the connections (Breeding et al. 1993). Preload is positively related to the screw‐tightening torque amounts (Huang and Wang 2019). An ideal preload is usually 60%–80% of the yield strength of the materials (Haack et al. 1995; McGlumphy et al. 1998; Piermatti et al. 2006; Siamos et al. 2002). Only 10% of the torque is converted into preload, while the remaining 90% is used for overcoming the friction between the operative connection surfaces (Piermatti et al. 2006; Siamos et al. 2002; Park et al. 2010). The loss of preload in the first 2–3 min (Assunção et al. 2011; Kano et al. 2006; Delben et al. 2011; Assunção et al. 2009) or 15 h (Wittneben et al. 2014) after tightening has been observed even without an external force.

By applying the torque force, the elastic recovery of the screw pulls the components together. Thus, a damping force is produced (Hotinski and Dudley 2019). In the design of a strong screw connection, the most important functional consideration is the primary damping force produced by tightening the screw (Siamos et al. 2002). The screw loosens when the external separating force applied on the implant‐abutment connection exceeds the damping forces that keep the implant and the abutment near each other (Piermatti et al. 2006; Winkler et al. 2003). Bickford (Bickford 1995). The prevalence of the loosening of the abutment screw is up to 7.12% in singular crowns and 7.6% in splinted ones (El‐Sheikh et al. 2018). Despite the numerous clinical and laboratory studies, the exact reason for abutment screw loosening remains unknown (Lee and Cha 2018). Insufficient torque force for tightening the screw, inadequate implant position, occlusal design with inadequate crown anatomy, frameworks with poor compatibility, microleakages in the implant‐abutment interface, inadequate screw design/material, and heavy occlusal force may be reasons for the loosening of implant screw (Sahin and Ayyildiz 2014; Cho et al. 2015; Tsumita et al. 2013; Binon 1996). Abutment screw loosening might lead to the mobility of the prosthesis, which needs removal of the prosthesis to tighten the abutment (Arshad et al. 2017).

Instability of the prosthesis, which occurs due to the loosening of the abutment screw, might change the distribution of occlusal force while functioning, therefore, the advance of the abutment loosening is accelerated (Xia et al. 2014; Fu et al. 2012). Also, abutment screw loosening increases micromotion and the micro gap in the implant‐abutment interface, thus exacerbating the microleakage in the implant‐abutment contact surface and finally causing biological complications (Sahin and Ayyildiz 2014; Broggini et al. 2003; Park 2008). Screw loosening leads to the instability of the implant‐abutment connection and the formation of microgap, which leads to the fracture of the components or the implant. This gap may cause the penetration of microorganisms, which is harmful to the surrounding membranes (Cássio do Nascimento et al. 2012). Also, loosening or fracture of the implant screws might lead to the fracture of the components, which leads to more expensive repairs (Taylor 1998; Stüker et al. 2008). Usually, simple tightening or abutment screw replacement is needed, but sometimes more repairs will be needed (Sakaguchi and Borgersen 1995). Therefore, the most common complications of abutment screw loosening include gingivitis and fracture of the screws (Zeno et al. 2016). To reduce these problems, different solutions have been suggested; including the use of diamond‐like carbon coating on the abutment screw, re‐tightening of the screw after the primary tightening, and increase of the torque amount (Siamos et al. 2002; Diez et al. 2012; Cho et al. 2004; Jung et al. 2008).

Bone‐level and tissue‐level implants are selected according to the clinical status of the patient and the decision of the dentist. It has to be considered that the difference in soft tissue histology is more related to the patient than the implant type. The conducted investigations indicate that neither implant type shows a significant difference based on clinical and histological parameters. Nevertheless, in tissue‐level implants, the collar part of the implant has shown a greater risk of remaining exposed (Menini et al. 2022). Continuous efforts have been made to increase the durability of implants, abutment screws, and implant‐abutment connections and to reduce problems concerning dental implants, and many studies have been done in this area but they lack (Attiah et al. 2020). Due to the limitations in investigations about the relation between the implant being new and the screw being old and the loosening and the torque reduction of the abutment screw in bone‐level and tissue‐level implants, this study has specifically been conducted on implants by DIO company.

## 2 Materials and Methods

**Figure 1:**
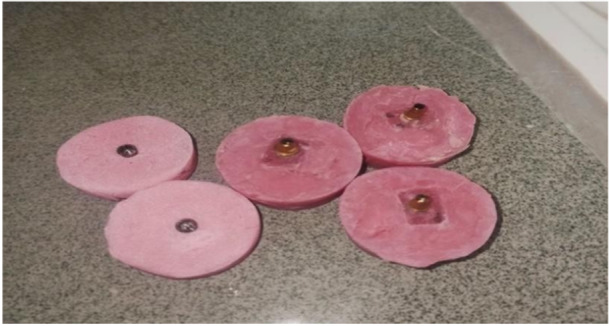
The samples of bone‐level and tissue‐level mounted implants.

This field study was conducted using a laboratory‐experimental method. The present study was conducted using a laboratory method in the Research Center, Faculty of Dentistry, Medical University, in 2024. This laboratory study includes every sample used in the laboratory process. According to the laboratory type of the study, every sample has been investigated. Thus, no specific criteria have been considered for the inclusion and exclusion of the samples. The sample size was calculated in the effect of cyclic dynamic loading on the loosening of tightened abutment screws, in comparison with the new screw in tissue‐level and bone‐level implants (laboratory study), at a significant level of 5% (α = 0.05), with the power of the test of 80% (β = 0.2), based on the study by Attiah et al. (2020), to diagnose a maximum difference equal to 9.1 times the standard deviation (= 9.1). About 20 implant‐abutment sets (regular) by DIO company, South Korea, were produced, having a diameter of 5.5 millimeters (regular). DIO Corp is the manufacturer of DIO implants. This implant was chosen because it is widely used in Iran. The minimum required sample size for all groups was calculated to be 40 specimens, 10 specimens for each group. Sample size calculations were performed using a software program (G*Power v3.0.1; Heinrich Heine University Düsseldorf). Half of these implants were bone‐level, and the other was tissue‐level. 10 new bone‐level implant‐abutment set screws (regular) and 10 new tissue‐level implant‐abutment set screws (regular) by DIO were provided. To mount the implant implants and simulate the bone tissue, blocks of acrylic resin with the dimensions of 6 mm × 10 mm × 20 mm were made. The manufactured implants were randomly mounted on these blocks (Figure 1). To check and confirm the vertical position of the implants inside the mentioned resinous blocks, a dental surveyor (Ney Surveyor, Ney Dental, Bloomfield, CT, USA) was used. In the next step, the implant‐abutment set was directly placed into the plastic block with the help of the analyzer bar, and self‐curing acrylic resin (Acropars, Iran) was injected around it, reaching below the polishing surface of the implant.

After that, the implants were torqued by a torque equal to 30 Newtons per centimeter. To apply and assess the torque amount, the DID‐4 torque meter by CEDAR was used. It has to be mentioned that 10 min after the first time applying the torque, the set was torqued again up to 30 Newtons per centimeter to prevent errors caused by the settling effect. To simulate the conditions of the oral environment in the samples, they were placed in each basket of the Delta Tpo2, Nemo thermocycler Research Center, Faculty of Dentistry, at 55 ± 5 degrees Celsius (to approximate the temperature of mouth while drinking hot and cold drinks), for 10,000 dynamic cycles, equal to 1 year, with the settling time of 20 s (Winkler et al. 2003; Gale and Darvell 1999). After that, the screws were opened, and the required torque force for opening the screws was recorded by the digital torque meter.

After applying the cycle and obtaining the values related to the 20 new screws, the same screws were used as screws to which dynamic cycles equal to 1 year were applied, and this time, the implant‐abutment sets were torqued up to 30 Newtons per centimeter by the torque meter, having old screws. Then, after 10 min, the sets were once again torqued up to 30 Newtons per centimeter and again placed in the thermocycler with the same conditions. After applying the cyclic dynamic cycle with the previous conditions, the opening torque of the screws was once again measured. To calculate the torque reduction amount, the following formula was used:

$$RTL=tightening\;torque-opening\;torque\times tightening\;torque_{100}$$

The present study is a laboratory study, and according to the fact that a device was needed to apply the thermodynamic cycle, the Delta Tpo2, Nemo thermocycler in Dr. Behrooz Mousavi's Building Research Center, Faculty of Dentistry of Isfahan was used, and since the torque amount and its changes were the main outcomes investigated in this study, the digital torque meter was the main tool used for measuring torque in the present study. The used torque meter was DID‐4 by CEDAR.

Based on the fact that a questionnaire was not used, the validity and reliability of the instruments make no sense in this study. To analyze the data, SPSSV26 software was used. The description of the quantitative variables was done through the average, and the standard deviation and description of the qualitative variables was done through the frequency and percentage. To evaluate the normal distribution of the data, the Kolmogorov–Smirnov test was used. The comparison of the torque reduction amounts between the two groups was made through Independent Samples *t*‐test. Also, the evaluation of the effect of the application of dynamic forces was made through the Paired Samples *t*‐test. The present study was conducted in a laboratory manner. It has to be mentioned that the moral considerations of this study were confirmed by the Ethics Committee of the Faculty of Dentistry of the Medical University of Isfahan by the code of IR.MUI.REC.1402.009.

## 3 Results

In the end, 10 samples were investigated in each group. In general, opening torque scores in the new bone‐level implants were more than in the new tissue‐level implants. Also, the torque reduction percentage values in the groups were compared, which showed that the torque reduction ratio in the tissue‐level group had greater values. The average required torque for opening the new tissue‐level implant after applying the primary thermodynamic cycle was 24.5 N cm, and in bone‐level implants, it was 26.5 N cm (Table 1).

**Table 1:** The opening torque values and the torque reduction percentage of the implants with new screws after applying the primary thermodynamic cycle (N cm).

Variable	Frequency	Average	Standard deviation
Required torque for opening the new tissue‐level implant after applying the primary thermodynamic cycle	10	24.7	0.65
Required torque for opening the new bone‐level implant after applying the primary thermodynamic cycle	10	26.7	0.62
Torque reduction percentage of the new tissue‐level implant after applying the primary thermodynamic cycle	10	17.67	2.17
Torque reduction percentage of the new bone‐level implant after applying the primary thermodynamic cycle	10	11	2.05

Also, the required torque amount and torque reduction percentage after applying the thermodynamic cycle were checked in the implants with old screws, which indicated lower opening torque amounts (23.23 N cm) and higher torque reduction amounts in the tissue‐level implants group (22.57 N cm) (Table 2).

**Table 2:** The opening torque values and the torque reduction percentage of the implants with old screws after applying the secondary thermodynamic cycle (N cm).

Variable	Frequency	Average	Standard deviation
Required torque for opening the new tissue‐level implant after applying the secondary thermodynamic cycle	10	23.23	0.39
Required torque for opening the new bone‐level implant after applying the secondary thermodynamic cycle	10	24.05	0.31
Torque reduction percentage of the new tissue‐level implant after applying the secondary thermodynamic cycle	10	22.57	1.32
Torque reduction percentage of the new bone‐level implant after applying the secondary thermodynamic cycle	10	19.83	1.05

The normal distribution of the data was evaluated based on the Kolmogorov–Smirnov test. The outcomes of statistical analysis indicated the normal distribution of all quantitative variables (Table 3).

**Table 3:** The outcomes of the Kolmogorov–Smirnov test on the opening torque amounts and torque reduction.

Variable	Statistic	*p* value
Opening torque of the implant with a new screw	0.124	0.200
Opening torque of the implant with an old screw	0.130	0.200
Torque reduction percentage of the implant with an old screw	0.031	0.200
Torque reduction percentage of the implant with a new screw	0.130	0.200

Also, the opening torque amounts and torque changes in bone‐level and tissue‐level implants were compared after applying the primary thermodynamic cycle, and the outcomes of the statistical analysis through the Independent Samples *t*‐test indicated that the opening torque amounts were significantly higher in the bone‐level group. It is obvious that the torque reduction percentage values of the implant were significantly higher in the tissue‐level group (Table 4).

**Table 4:** Comparison of the opening torque amounts and torque reduction percentage in the implants with new screws after applying the primary thermodynamic cycle, based on the implant type (N cm).

Variable	Bone‐level		Tissue‐level		*p* value
	Average	Standard deviation	Average	Standard deviation	
Required torque for opening the implant after applying the primary thermodynamic cycle	26.7	0.62	24.7	0.39	0.000
The torque reduction percentage of the implant after the primary thermodynamic cycle	11	2.05	17.67	2.17	0.000

After applying the secondary thermodynamic cycle, the torque amounts and torque amount reduction were compared between the two groups of bone‐level and tissue‐level implants, and the outcomes of the statistical analysis through the Independent Samples *t*‐test indicated higher opening torque amounts in the bone‐level group. Also, the implant torque reduction percentage was lower in the bone‐level group (Table 5).

**Table 5:** Comparison of the opening torque amounts and torque reduction percentage of the implants with old screws after applying the secondary thermodynamic cycle, based on the implant type (N cm).

Variable	Bone‐level		Tissue‐level		*p* value
	Average	Standard deviation	Average	Standard deviation	
Required torque for opening the implant after applying the dynamic cycle	24.05	0.65	2.23	0.31	0.000
The torque reduction percentage of the implant after applying the dynamic cycle	19.83	1.05	22.57	1.32	0.000

**Figure 2:**
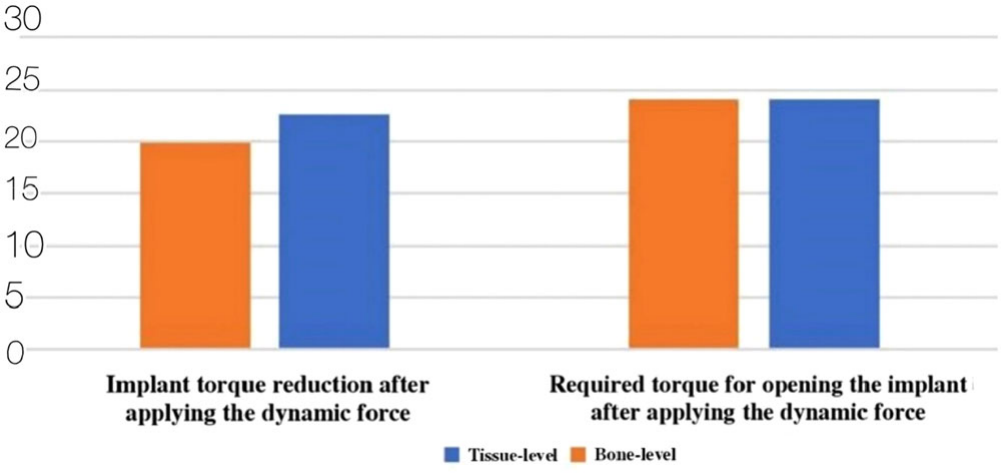
Comparison of the opening torque amounts and torque reduction percentage of the implants with new screws after applying the secondary thermodynamic cycle, based on the implant type (N cm).

Also, the impact of applying the primary and secondary thermodynamic cycles on the opening force amount and torque reduction percentage was evaluated in each of the bone‐level and tissue‐level groups (Figure 2).

**Figure 3:**
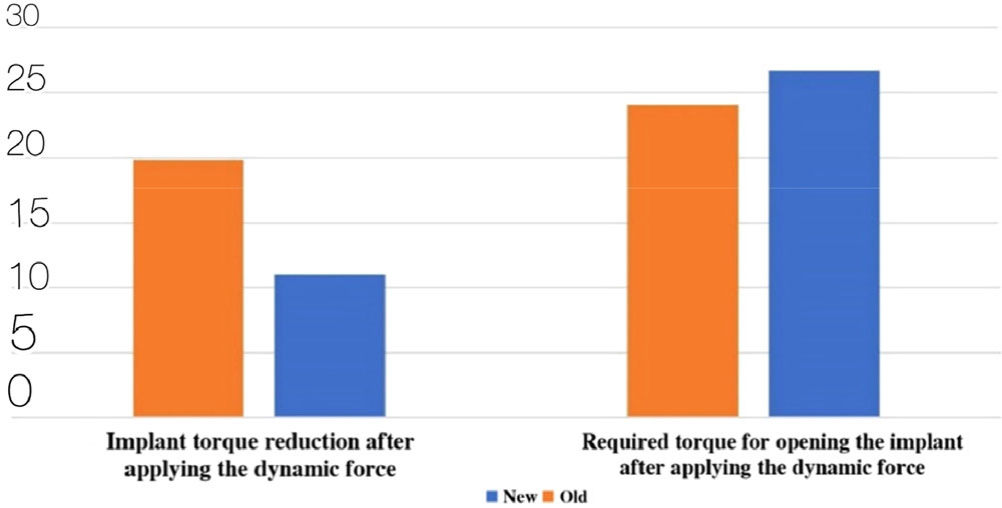
Comparison of the opening torque amounts and torque reduction percentage of the bone‐level implants after applying the primary dynamic cycle and after applying the secondary dynamic cycle (N cm).

In the bone‐level implants group, the torque force amount decreased significantly after applying the secondary thermodynamic cycle, compared with the primary cycle. Also, the results of statistical analysis through the Paired Samples *t*‐test indicated a significant increase in torque reduction as an effect of the cycle application. (Table 6) (Figure 3).

**Table 6:** Comparison of the opening torque amounts and torque reduction percentage of the bone‐level implants after applying the primary dynamic cycle and after applying the secondary dynamic cycle (N cm).

Variable	New		Old		*p* value
	Average	Standard deviation	Average	Standard deviation	
Required torque for opening the implant	26.7	0.62	24.05	0.31	0.000
Implant torque reduction percentage	11	2.05	19.83	0.05	0.000

**Figure 4:**
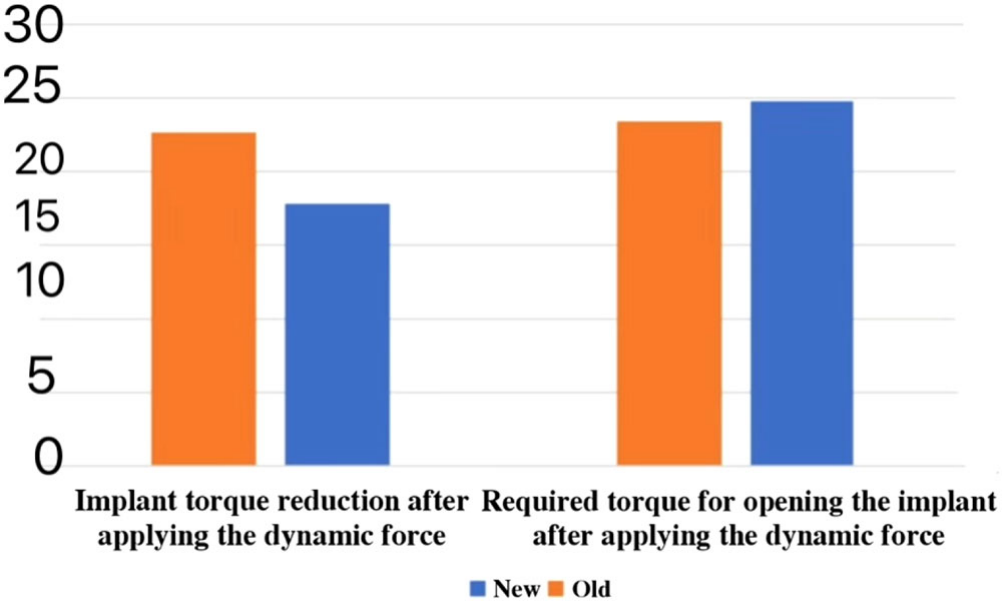
Comparison of the opening torque amounts and torque reduction percentage of the tissue‐level implants after applying the primary dynamic cycle and after applying the secondary dynamic cycle (N cm).

Also, in the tissue‐level implants group, the opening force amount after applying the secondary cycle decreased significantly in comparison with the primary cycle, and also, the statistical analysis through the Paired Samples *t*‐Test indicated a significant increase in the torque reduction percentage value in this group of implants (Table 7) (Figure 4).

**Table 7:** Comparison of the opening torque amounts and torque reduction percentage of the tissue‐level implants after applying the primary dynamic cycle and after applying the secondary dynamic cycle (N cm).

Variable	New		Old		*p* value
	Average	Standard deviation	Average	Standard deviation	
Required torque for opening the implant	24.7	0.65	23.23	0.39	0.000
Implant torque reduction	17.67	2.17	22.57	1.32	0.000

## 4 Discussion

The outcomes of this laboratory study indicate the impact of applying a dynamic cycle on the torque and also the torque reduction of the implant screws, regardless of the implant type. Screw loosening of the implants is a common mechanical complication that may occur in implant‐based restorations. This incident is the most common mechanical problem that dentists face, and its incidence varies from 2 to 45 percent, depending on the restoration type (Alsubaiy 2020; Gupta et al. 2015). Various factors can play a role in the loosening of the screws, including the screw length, materials, and design and also the quality of the implant‐abutment connection (Alsubaiy 2020; Halevy‐Politch 2024). The posterior implants are more likely to suffer screw loosening in comparison with the anterior implants, most likely due to higher occlusal forces (Gupta et al. 2015). To prevent screw loosening, the dentists must make sure of proper implant placement, use components with strong tolerances and anti‐rotary characteristics, and continuously tighten the screws to the recommended torques using mechanical torque gauges (Alsubaiy 2020; Gupta et al. 2015).

Nevertheless, implant screw loosening is still a prevalent incident. Generally, the screws loosen due to the application of bite forces and the dynamic intraoral conditions. The current solution in this situation is tightening the loosened screw. However, as it was indicated, the resultant forces might lead to torque reduction of the screws, and therefore disfunction of them. Thus, replacement of the screw might be considered as a solution. As it was mentioned, the application of dynamic cycles increases the torque reduction amounts in the implants. In general, it is assumed that the application of dynamic cycles has a significant impact on screw loosening in dental implants, which is mainly because of the mechanical tensions applied to the screws during functional loading. Although the results of a recent laboratory study have indicated that after applying 100,000 dynamic cycles of loading (DCL), the re‐tightened abutment screws and the new screws showed different grades of rupture torque loss (RTL), and higher amounts were observed in implants with narrower diameters than in standard implants. In addition to the results, a recent study showed that re‐tightening the existing screws usually has better torque maintenance than replacing them with new screws, which shows that frequent tightening might increase the screw stability by reducing the surface harshnesses and improving the friction between the screw and the implant interface (Attiah et al. 2020).

Generally, dynamic cycles can lead to rupture torque loss (RTL) in dental implants, which is usually due to mechanical fatigue and micromotions because of frequent loadings. When implants are exposed to dynamic cycles of loading, they suffer forces that might lead to subtle movements in the implant‐abutment connection. These extremely small movements can, over time, reduce the preload of the screws which is essential for maintaining their stability (Ricciardi Coppedê et al. 2009). The other finding of this study was that the lower opening torque amounts were in tissue‐level implants. Also, the torque reduction amount was higher in this type of implant. The studies show that tissue‐level implants usually have more torque reduction in comparison than bone‐level implants. Also, a recent study has shown that the average percentage of torque reduction for implants with narrower diameters was more than the standard implants, which shows that the design and position of the implant can significantly affect the stability of the screw under the conditions of dynamic loading. Also, these amounts were less in this type of implant after force application. In general, after being exposed to the application of dynamic forces, the loss of torque after loading is significantly greater for tissue‐level implants. This issue exists due to the higher mechanical stresses that these implants experience while functional loading, which can lead to abutment screw loosening (Attiah et al. 2020).

Clinical studies are needed to validate the outcomes observed in this study and provide insights for real‐world applications.

### 4.1 Limitations

Limited sample size and manufacturer bias were the limitations of our study.

## 5 Conclusion

The outcomes of this study indicate that to fix the problem of implant screw loosening, replacement of the old screw with a new screw may be effective in preventing it from loosening again. Also, the results of this study indicate the higher importance of this phenomenon in screw loosening of tissue‐level implants.

## Author Contributions

**Amirhossein Fathi:** conceptualization, methodology, writing – original draft, investigation. **Sina Borhani:** validation, formal analysis. **Sepideh Salehi:** writing – review and editing, supervision, software. **Ramin Mosharraf:** visualization, resources. **Ramin Atash:** project administration.

## 1 Conflicts of Interest

The authors declare no conflicts of interest.

## Data Availability

Data would be available upon request due to ethical restrictions.
